# Micromagnetic simulation of exchange coupled ferri-/ferromagnetic heterostructures

**DOI:** 10.1016/j.jmmm.2014.12.045

**Published:** 2015-05-01

**Authors:** Harald Oezelt, Alexander Kovacs, Franz Reichel, Johann Fischbacher, Simon Bance, Markus Gusenbauer, Christian Schubert, Manfred Albrecht, Thomas Schrefl

**Affiliations:** a Industrial Simulation, St. Pölten University of Applied Sciences, Matthias Corvinus-Straße 15, A-3100 St. Pölten, Austria; b Institute of Physics, Chemnitz University of Technology, Reichenhainer Straße 70, D-09126 Chemnitz, Germany; c Institute of Physics, University of Augsburg, Universitätsstraße 1, D-86159 Augsburg, Germany; d Center for Integrated Sensor Systems, Danube University Krems, Viktor Kaplan-Straße 2, A-2700 Wiener Neustadt, Austria

**Keywords:** Exchange-coupled composite media, Micromagnetic simulations, Ferrimagnet, FEM, Domain wall motion, Finite size effect, Pinning

## Abstract

Exchange coupled ferri-/ferromagnetic heterostructures are a possible material composition for future magnetic storage and sensor applications. In order to understand the driving mechanisms in the demagnetization process, we perform micromagnetic simulations by employing the Landau–Lifshitz–Gilbert equation. The magnetization reversal is dominated by pinning events within the amorphous ferrimagnetic layer and at the interface between the ferrimagnetic and the ferromagnetic layer. The shape of the computed magnetization reversal loop corresponds well with experimental data, if a spatial variation of the exchange coupling across the ferri-/ferromagnetic interface is assumed.

## Highlights

• We present a model for exchange coupled ferri-/ferromagnetic heterostructures.
• We incorporate the microstructural features of the amorphous ferrimagnet.
• A distribution of interface exchange coupling is assumed to fit experimental data.
• The reversal is dominated by pinning within the ferrimagnet and at the interface.

## 1 Introduction

Ferrimagnetic materials have been widely used as magneto-optical recording media [1,2] and provide great potential for future devices in sensor technology and magnetic recording. Interest in ferrimagnetic materials has been renewed by experiments revealing all-optical switching of the magnetization [3–5] and the building of heterostructures leading to giant exchange bias [6]. Further applications of heterostructures built on ferromagnetic and ferrimagnetic layers might be found in magnetic recording media with tailored switching behaviour. The big advantage of these materials is the ability to tailor their magnetic properties by their composition with respect to the desired working temperature [7]. In order to exploit these properties we not only need to gain a deeper understanding of such materials but also need to investigate the exchange coupling with ferromagnetic materials. Exchange coupled composites (ECC) of hard- and soft-magnetic phases have already been proposed for the next generation of magnetic recording media [8] and may very well benefit even more from tailored ferrimagnetic layers.

Ferrimagnetic thin films have been extensively studied by Giles and Mansuripur et al. [9–12] in terms of magneto-optical recording. In their work they investigated the magnetization reversal dynamics and domain wall motion by utilizing an adapted Gilbert equation on a two dimensional lattice of magnetic dipoles. We will later use this approach in a three dimensional model system of ferrimagnetic thin films (see Section 2.1).

Yamada and his collaborators [13] experimentally showed the approach of using an exchange coupled magnetic capping layer on a ferrimagnetic layer (TbFeCo) to lower the required external field for magneto-optical recording. In contrast to our simulations the used capping layer had in-plane magnetization.

In experiments with strongly exchange coupled TbFe/FeCo multilayers, Armstrong et al. [14] revealed that demagnetization occurs by nucleation of a domain which extends through the entire layer-stack. A single twin wall is formed which moves until the whole sample is reversed. Contrary to our investigated model the layers are coupled antiparallel and have an in-plane easy axis.

Antiferromagnetically exchange coupled ferri-/ferrimagnetic bilayers have been investigated by Mangin et al. [15]. In their work they identified the magnetic configuration at the interface as the determining mechanism for the exchange bias field.

A general micromagnetic model for exchange coupled bilayer systems was described by Oti [16]. He simulated laminated cobalt-alloy films used in longitudinal recording. The effect of media dimensions and interface exchange on magnetization at remanent and coercive states for two layers separated by a nonmagnetic phase were investigated. Both layers are modelled as an array of uniaxial volume elements and show an isotropic three-dimensional distribution of magnetocrystalline anisotropy axes.

Schubert and collaborators [17] experimentally investigated the interface exchange coupling of ferri-/ferromagnetic heterostructures with out-of-plane anisotropy. Their results revealed that an interfacial domain wall greatly affects the demagnetization process.

In this paper we start by taking Mansuripur's approach [11] for ferrimagnetic films with strongly coupled sub-lattices and implement it in a three-dimensional micromagnetic finite element calculation. We extend the model by adding a collinearly exchange-coupled continuous ferromagnetic layer. The magnetization reversal of this bilayer system is compared with recently accomplished experimental results [17]. Finally the demagnetization process is studied by visualizing and comparing the movement of domain walls through a single ferrimagnetic layer and a ferri-/ferromagnetic bilayer system.

## 2 The model system

### 2.1 Micromagnetic model for ferrimagnetic thin films

While the finite element simulation of ferromagnets is a common task, ferrimagnets have been simulated on their own and in two dimensions for the application in magneto-optical recording [12,18]. Ferrimagnets have different sublattices with unequal opposing magnetic moments, hence the mathematical model has to be adapted. By following Mansuripur [11], assuming that the sublattices are strongly coupled antiparallel, the usual Gilbert equation can be written for each of sublattices $L^{\left(a\right)}$ and $L^{\left(b\right)}$ as

(1a) $${\overset{.}{M}}^{\left(a\right)}=-\gamma^{\left(a\right)}M^{\left(a\right)}\times (H^{\left(a\right)}+hM^{\left(b\right)})+\alpha^{\left(a\right)}M^{\left(a\right)}\times {\overset{.}{m}}^{\left(a\right)}$$

(1b) $${\overset{.}{M}}^{\left(b\right)}=-\gamma^{\left(b\right)}M^{\left(b\right)}\times (H^{\left(b\right)}+hM^{\left(a\right)})+\alpha^{\left(b\right)}M^{\left(b\right)}\times {\overset{.}{m}}^{\left(b\right)}$$

**Fig. 1:**
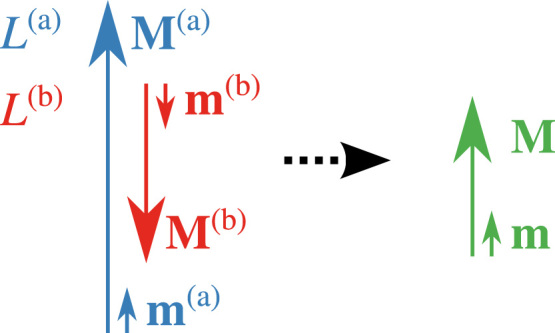
In this model the magnetic moments $M^{\left(a\right)}$ and $M^{\left(b\right)}$ of the sublattices $L^{\left(a\right)}$ and $L^{\left(b\right)}$ of a ferrimagnet are assumed to be antiparallel at all times and therefore substituted by an effective net magnetization M.

The sublattice $L^{\left(a\right)}$ is defined by its magnetization magnitude $M^{\left(a\right)}$ and its unit vector $m^{\left(a\right)}=M^{\left(a\right)}/M^{\left(a\right)}$, the gyromagnetic ratio $\gamma^{\left(a\right)}$ and the corresponding damping parameter $\alpha^{\left(a\right)}$. The field on the subnet $L^{\left(a\right)}$ is split into the effective local exchange field $hM^{\left(b\right)}$ of subnet $L^{\left(b\right)}$ acting on subnet $L^{\left(a\right)}$ and the remaining effective fields $H^{\left(a\right)}$. This notation applies to the sublattice $L^{\left(b\right)}$. *h* is the effective coupling constant between the sublattices. Because of reciprocity of the exchange energy, *h* is the same for each sublattice. Due to the strongly coupled sublattices, the magnetic moments $M^{\left(a\right)}$ and $M^{\left(b\right)}$ will always stay antiparallel. Therefore the effective net magnetization can be defined as M=Mm with $M=M^{\left(a\right)}-M^{\left(b\right)}$ and $m=m^{\left(a\right)}=-m^{\left(b\right)}$ (see Fig. 1).

By summing up Eqs. (1a) and (1b) and substituting the unit vectors, we achieve

(2) $$(\frac{M^{\left(a\right)}}{\gamma^{\left(a\right)}}-\frac{M^{\left(b\right)}}{\gamma^{\left(b\right)}})\overset{.}{m}=-m\times (M^{\left(a\right)}H^{\left(a\right)}-M^{\left(b\right)}H^{\left(b\right)})\;+(\frac{\alpha^{\left(a\right)}M^{\left(a\right)}}{\gamma^{\left(a\right)}}+\frac{\alpha^{\left(b\right)}M^{\left(b\right)}}{\gamma^{\left(b\right)}})m\times \overset{.}{m}$$

By defining the effective values as

(3) $$\gamma_{\mathrm{eff}}=\frac{M^{\left(a\right)}-M^{\left(b\right)}}{\frac{M^{\left(a\right)}}{\gamma^{\left(a\right)}}-\frac{M^{\left(b\right)}}{\gamma^{\left(b\right)}}}$$

(4) $$\alpha_{\mathrm{eff}}=\frac{\frac{\alpha^{\left(a\right)}M^{\left(a\right)}}{\gamma^{\left(a\right)}}+\frac{\alpha^{\left(b\right)}M^{\left(b\right)}}{\gamma^{\left(b\right)}}}{\frac{M^{\left(a\right)}}{\gamma^{\left(a\right)}}-\frac{M^{\left(b\right)}}{\gamma^{\left(b\right)}}}$$

(5) $$H_{\mathrm{eff}}=\frac{M^{\left(a\right)}H^{\left(a\right)}-M^{\left(b\right)}H^{\left(b\right)}}{M^{\left(a\right)}-M^{\left(b\right)}}$$

the Gilbert equation of a strongly coupled ferrimagnetic thin film is obtained:

(6) $$\overset{.}{m}=-\gamma_{\mathrm{eff}}m\times H_{\mathrm{eff}}+\alpha_{\mathrm{eff}}m\times \overset{.}{m}$$

We particularize Eq. (5) by splitting the effective fields $H^{\left(a\right)}$ and $H^{\left(b\right)}$ into a sum of their components: the external field $H_{\mathrm{ext}}$, the demagnetizing field $H_{\mathrm{dmag}}$, the anisotropy field $H_{\mathrm{ani}}$ and the exchange field $H_{\mathrm{xhg}}$. The external field and the demagnetizing field are equal for both subnets:

(7) $$H_{\mathrm{dmag}}^{\left(a\right)}=H_{\mathrm{dmag}}^{\left(b\right)}=H_{\mathrm{dmag}}$$

(8) $$H_{\mathrm{ext}}^{\left(a\right)}=H_{\mathrm{ext}}^{\left(b\right)}=H_{\mathrm{ext}}$$

For the anisotropy field we assume a common anisotropic easy axis, defined by an unit vector k, but different magnetic anisotropy constants $K_{u}^{\left(a\right)}$ and $K_{u}^{\left(b\right)}$:

(9a) $$H_{\mathrm{ani}}^{\left(a\right)}=\frac{2K_{u}^{\left(a\right)}}{M^{\left(a\right)}}(m^{\left(a\right)}\cdot k)k$$

(9b) $$H_{\mathrm{ani}}^{\left(b\right)}=\frac{2K_{u}^{\left(b\right)}}{M^{\left(b\right)}}(m^{\left(b\right)}\cdot k)k$$

The exchange fields have different exchange constants $A_{x}^{\left(a\right)}$ and $A_{x}^{\left(b\right)}$ and are proportional to the Laplacian of their respective magnetization:

(10a) $$H_{\mathrm{xhg}}^{\left(a\right)}=\frac{2A_{x}^{\left(a\right)}}{{M^{\left(a\right)}}^{2}}\nabla^{2}M^{\left(a\right)}$$

(10b) $$H_{\mathrm{xhg}}^{\left(b\right)}=\frac{2A_{x}^{\left(b\right)}}{{M^{\left(b\right)}}^{2}}\nabla^{2}M^{\left(b\right)}$$

We define an effective net anisotropy constant $K_{u}=K_{u}^{\left(a\right)}+K_{u}^{\left(b\right)}$ and an effective net exchange stiffness constant $A_{x}=A_{x}^{\left(a\right)}+A_{x}^{\left(b\right)}$ and rewrite Eq. (5) with $M=M^{\left(a\right)}-M^{\left(b\right)}$ as follows:

(11) $$H_{\mathrm{eff}}=H_{\mathrm{ext}}+H_{\mathrm{dmag}}+\underset{H_{\mathrm{ani}}}{\underset{︸}{\frac{2K_{u}}{M}(m\cdot k)k}}+\underset{H_{\mathrm{xhg}}}{\underset{︸}{\frac{2A_{x}}{M}\nabla^{2}m}}$$

Eqs. (3), (4) and (11) represent the parameters for the Gilbert equation (6) which we solve by employing the finite element micromagnetic package *FEMME* [19,20].

**Fig. 2:**
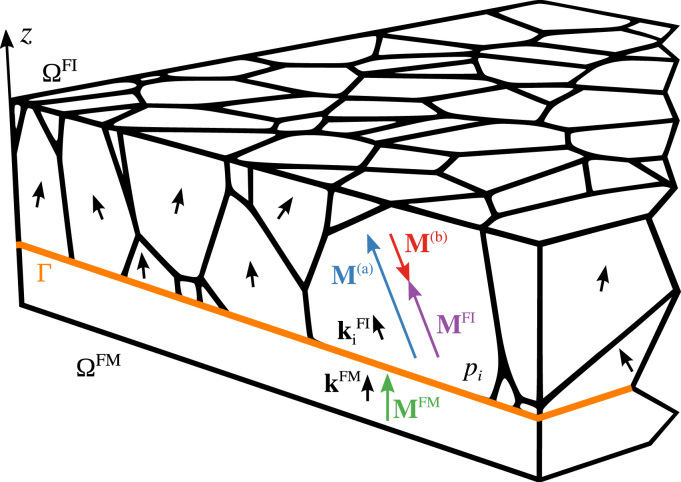
Geometric model of a bilayer system with a ferrimagnetic phase $\Omega^{\mathrm{FI}}$ and a ferromagnetic phase $\Omega^{\mathrm{FM}}$ connected at the interface Γ. The amorphous $\Omega^{\mathrm{FI}}$ is separated in regions (patches *p*_*i*_) with varying uniaxial anisotropic direction $k_{i}^{\mathrm{FI}}$ and anisotropic constant $K_{\;u,i}^{\;\mathrm{FI}}$. $\Omega^{\;\mathrm{FM}}$ is a continuous phase with a weak out-of-plane uniaxial anisotropy $k^{\mathrm{FM}}$.

As Giles et al. suggested in [21,9,12], real amorphous rare earth-transition metal alloys show increased coercivity, probably due to spatial fluctuations of magnetic properties and material inhomogeneities. In order to become effective, these properties have to be distributed over patches with at least the size of the domain wall width. This structural property might be attributed to a still existing near-range order in the amorphous material. Based on [12], we consider a spatial distribution of the magnetocrystalline anisotropy axis and the anisotropy constant. Therefore these two parameters, defining the anisotropic field in Eq. (11), are functions of space and become $k=k^{\mathrm{FI}}(x)$ and $K_{u}=K_{u}^{\mathrm{FI}}(x)$. From now on we use the superscript FI to denote the ferrimagnetic phase and FM for the ferromagnetic phase. The thin film model is divided into patches *p*_*i*_ with an average diameter of 13 nm (see upper layer $\Omega^{\mathrm{FI}}$ in Fig. 2). Both anisotropic properties are then randomly and independently distributed across these patches. This means that every patch *p*_*i*_ has its own $k_{i}^{\mathrm{FI}}$ and $K_{u,i}^{\mathrm{FI}}$. In order to model these patches we use a three-dimensional tetrahedron finite-element mesh created by the software package *NEPER* [22], which uses *Gmsh* [23] as its FE-mesh generator. This software allows us to create the patches by using Voronoi tessellation. The deviation of $k_{i}^{\mathrm{FI}}$ from the *z*-axis is limited by a maximum angle of $\theta_{\max}=\pi /4$ to preserve an overall out-of-plane anisotropy. The magnetic anisotropy constant varies with a standard deviation of $\sigma_{K}=0.2\langle K_{u}^{\mathrm{FI}}\rangle$. We especially want to point out that no other intrinsic properties differ from patch to patch.

### 2.2 Coupled ferri-/ferromagnetic bilayers

As the magnetic progression of a ferrimagnetic film can now be calculated, a collinearly exchange coupled ferromagnetic layer is added. The bilayer system with its ferrimagnetic phase $\Omega^{\mathrm{FI}}$, ferromagnetic phase $\Omega^{\mathrm{FM}}$ and interface Γ is depicted in Fig. 2. In order to model the exchange coupling at the interface we have to take into account the effective exchange field from the ferrimagnetic layer $H_{\mathrm{ixhg}}^{\mathrm{FI}}$ acting on $\Omega^{\mathrm{FM}}$ and the effective exchange field from the ferromagnetic layer $H_{\mathrm{ixhg}}^{\mathrm{FM}}$ acting on $\Omega^{\mathrm{FI}}$. Therefore we extend the equation for the effective fields of both layers, $\Omega^{\mathrm{FI}}$ and $\Omega^{\mathrm{FM}}$, as follows:

(12a) $$H_{\mathrm{eff}}^{\mathrm{FI}}=H_{\mathrm{ext}}+H_{\mathrm{dmag}}+H_{\mathrm{ani}}^{\mathrm{FI}}+H_{\mathrm{xhg}}^{\mathrm{FI}}+H_{\mathrm{ixhg}}^{\mathrm{FM}}$$

(12b) $$H_{\mathrm{eff}}^{\mathrm{FM}}=H_{\mathrm{ext}}+H_{\mathrm{dmag}}+H_{\mathrm{ani}}^{\mathrm{FM}}+H_{\mathrm{xhg}}^{\mathrm{FM}}+H_{\mathrm{ixhg}}^{\mathrm{FI}}$$

For the simulation both layers are separately represented by a three-dimensional tetrahedron finite-element (FE) mesh with a characteristic mesh size of 3 nm. Exchange coupling only affects spins within the exchange length (∼2.5 nm), hence the interface exchange field acts only on the mesh nodes at the interface of the two layers. The interfacial exchange fields can be calculated by the variation of the interface exchange energy $E_{\mathrm{ixhg}}$ over the magnetic moment as expressed in Eq. (14):

(13) $$H_{\mathrm{ixhg}}^{\mathrm{FI}}=H_{\mathrm{ixhg}}^{\mathrm{FM}}=0\;\mathrm{on}\;(\Omega^{\mathrm{FI}}\cup \Omega^{\mathrm{FM}})\backslash \Gamma$$

(14) $$\begin{array}{c} H_{\mathrm{ixhg}}^{\mathrm{FI}}=-\frac{1}{\mu_{0}}\frac{\delta E_{\mathrm{ixhg}}}{\delta M^{\mathrm{FI}}}\\ H_{\mathrm{ixhg}}^{\mathrm{FM}}=-\frac{1}{\mu_{0}}\frac{\delta E_{\mathrm{ixhg}}}{\delta M^{\mathrm{FM}}} \end{array}\}\;\mathrm{on}\;\Gamma$$

The exchange energy across the interface is given by

(15) $$E_{\mathrm{ixhg}}=-J_{\Gamma }\underset{i,j}{\sum }S_{i}^{\mathrm{FM}}S_{j}^{\mathrm{FI}}≃-\frac{J_{\Gamma }S^{2}}{a^{2}}\int_{\Gamma }u_{i}^{\mathrm{FM}}u_{j}^{\mathrm{FI}}\;d\Gamma \;=-J_{\mathrm{ixhg}}\int_{\Gamma }u_{i}^{\mathrm{FM}}u_{j}^{\mathrm{FI}}\;d\Gamma$$

where $J_{\Gamma }$ is the exchange integral, $S_{i}^{\mathrm{FM}}$ and $S_{j}^{\mathrm{FI}}$ are the respective spins, *a* is the distance of the spins in a simple cubic lattice and $J_{\mathrm{ixhg}}$ is the interface exchange coupling constant. In Eq. (15) the transition from a discrete spin model to a continuous description with the unit magnetization vectors $u_{i}^{\mathrm{FM}}$ and $u_{j}^{\mathrm{FI}}$ is made. In order to take into account the microstructural features of the ferrimagnet, as explained earlier in Section 2.1, the two layers have to be meshed separately. Hence the nodes at the interface of the two meshes do not match. This problem has been addressed in the study from Dean and his collaborators on antiferromagnetic/ferromagnetic bilayers [24] and therefore can be solved in the same manner. They employed a surface integral technique to restore the continuity at the interface and calculate $E_{\mathrm{ixhg}}$ using a symmetric Gaussian quadrature rule for triangles [25,26].

To be able to compare the simulation results with experimental data, we developed a model close to the ${\mathrm{Fe}}_{81}{\mathrm{Tb}}_{19}(20\;\mathrm{nm})$ layer exchange coupled to a ${\left[\mathrm{Co}(0.4\;\mathrm{nm})/\mathrm{Pt}(0.8\;\mathrm{nm})\right]}_{10}$ multilayer stack from the work of Schubert et al. [17]. The FeTb layer experimentally shows an out-of-plane anisotropy and represents the hard magnetic part of this bilayer system. This ferrimagnetic phase is modelled by a 100×100×20 nm layer of 120 patches with 13 nm average diameter ($\Omega^{\mathrm{FI}}$ in Fig. 2). In this particular system the Co/Pt multilayer stack of the experiment is modelled as a 12 nm thick continuous, soft magnetic layer $\Omega^{\mathrm{FM}}$ which is collinearly coupled to the ferrimagnet. The exchange constant $A_{x}$, the local uniaxial anisotropy constant $K_{u}$ and the effective saturation polarization $J_{s}=\mu_{0}M_{s}$ are listed in Table 1. For comparison with the data from [17] we choose the intrinsic properties at 70 K. The demagnetization curve at 70 K shows a well pronounced tail at high external field. In the remainder of the paper we will show that this tail can be attributed to a distribution of the interlayer coupling strength.

## 3 Results and discussion

### 3.1 Magnetization reversal of a ferri-/ferromagnetic bilayer

**Fig. 3:**
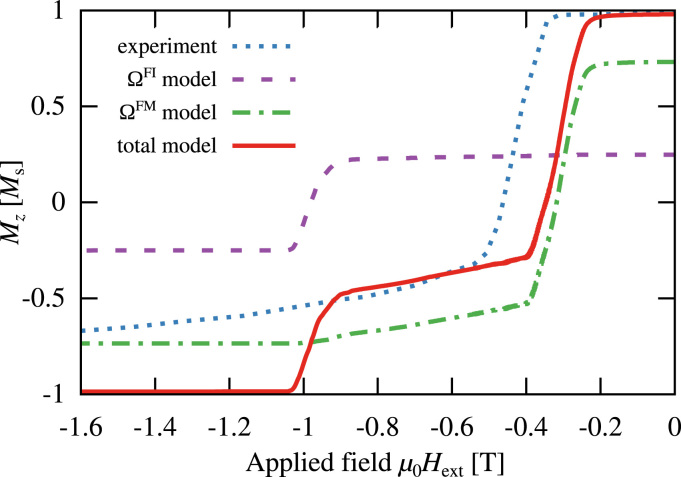
Computed demagnetization curves of the ferrimagnetic layer, the ferromagnetic layer and the overall system compared to the experimental measurement of Schubert et al. [17] at 70 K, all normalized by the total saturation magnetization $M_{s}$. The simulation was done with an interface exchange strength of $J_{\mathrm{ixhg}}=10\;\mathrm{mJ}/m^{2}$.

By applying an out-of-plane external field $H_{\mathrm{ext}}$ (parallel to the *z*-axis) the magnetization reversal curve of the bilayer system is computed. In the simulations we are interested only in the static hysteresis behaviour. Therefore we use an effective damping constant $\alpha_{\mathrm{eff}}=1$ and change the external field at the rate of 27 mT/ns, assuming an effective gyromagnetic ratio of $\gamma_{\mathrm{eff}}=0.63\;m/(\mathrm{sA})$ [11]. Fig. 3 shows the computed reversal curve of both layers separately and in total, all normalized by the total saturation magnetization. Additionally the experimentally measured curve from [17] is drawn. The soft magnetic $\Omega^{\mathrm{FM}}$ phase starts to switch at −0.25 T already, but due to interface exchange coupling the reversal process gets stopped close to the interface. The reversed domain approaches the interface and the domain wall pushes through when eventually the $\Omega^{\mathrm{FI}}$ phase switches at once at −0.9 T.

While the switching of the soft magnetic phase and reversal process at the interface matches the experimental measurement very well, the simulated ferrimagnetic phase reaches its negative saturation at a much lower field than in the experiment. This disagreement can be identified as finite size effect of the simulation, since the small model size cannot correctly represent the effect of a distribution of the exchange coupling strength across the interface of the much bigger measured sample. This effect can be overcome by averaging over many computed magnetization reversal curves for the same model but with different values of the interface exchange coupling constant $J_{\mathrm{ixhg}}$.

**Fig. 4:**
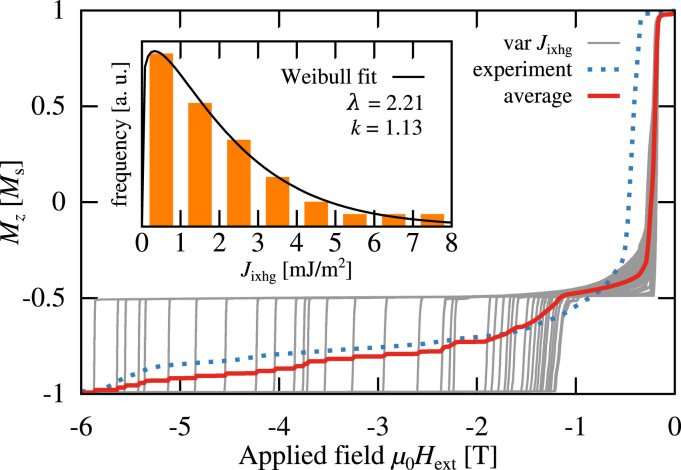
Averaged magnetization reversal curve (solid line) of the bilayer system compared to the experimental measurement (dotted line) of Schubert et al. [17]. The average is computed over a variation of simulation runs (bright solid lines) with an $J_{\mathrm{ixhg}}$-distribution shown in the inset.

We performed 40 simulations with different $J_{\mathrm{ixhg}}$. The values of $J_{\mathrm{ixhg}}$ were distributed randomly. The optimum distribution was found by repeated calculations of the averaged demagnetization curve. The distribution was adjusted manually in order to reduce the squared distance between the experimental demagnetization curve and the computed curve. The inset of Fig. 4 shows the distribution that minimizes the squared distance of the computed hysteresis loop to the experimental one. The frequency is fitted to a Weibull-distribution (16), which is also used to describe particle size distributions:

(16) $$f(x;\lambda ,k)=\frac{k}{\lambda }{\left(\frac{x}{\lambda }\right)}^{k-1}e^{-{\left(x/\lambda \right)}^{k}},\;\forall x\geq 0$$

The best fit is obtained for λ=2.21 and *k*=1.13. Fig. 4 shows the resulting average curve which reproduces the shape of the measured reversal curve.

We expect the distribution to change with temperature. Schubert and co-workers [17] show that the tail in the hysteresis loop changes with temperature and becomes longer with decreasing temperature. According to the model presented above, this would indicate that the width of the distribution increases with decreasing temperature. The fitted distribution shows that large portions of the interface area are weakly coupled and therefore favour the formation of an interface domain wall. Nucleation of patches in the ferrimagnetic layer occur first on those interface sites with strong exchange coupling. With increasing external field the reversal proceeds by pushing the domain wall in the ferrimagnetic phase from patch to patch towards full reversal. This movement of the domain wall is further investigated in Section 3.2.

**Fig. 5:**
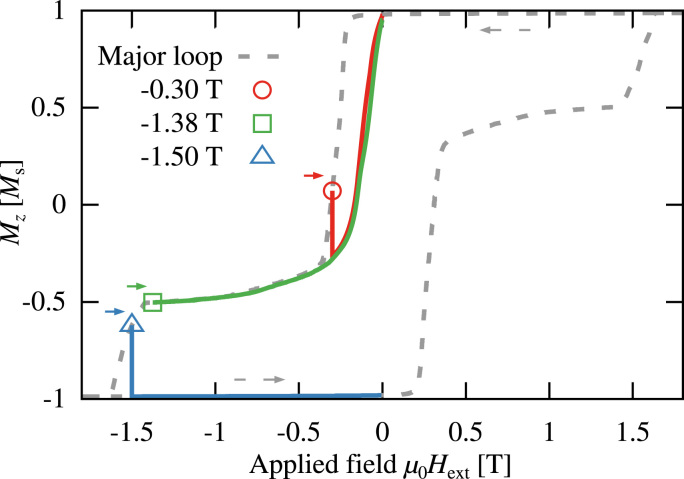
Minor reversal curves from a bilayer system with $J_{\mathrm{ixhg}}=14\;\mathrm{mJ}/m^{2}$. The two magnetization configurations, ◯ and □, are fully reversible. The ▵ curve, which starts after nucleation in the ferrimagnet, shows a changed remanent state.

Calculated minor reversal curves of the bilayer system are shown in Fig. 5. The minor curves starting at −0.3 T and −1.38 T show that the switching of the ferromagnet is fully reversible. As soon as the domain wall has been pushed into the ferrimagnetic phase by the applied field, irreversible switching of the ferrimagnet occurs and the reversal curve does not go back to remanence. The same result has been observed in [17], but with intermediate states where the Fe/Tb layer is not fully switched. The absence of this states can again be attributed to the $J_{\mathrm{ixhg}}$-distribution, as explained earlier in this section, which was not considered in this simulation.

### 3.2 Domain wall motion

Domain wall motion influences the magnetization reversal process in the ferri-/ferromagnetic bilayer. Here we study domain wall pinning and domain wall motion in a single FeTb layer (Section 3.2.1) and in a FeTb/CoPt bilayer system (Section 3.2.2). We artificially switch half of the ferrimagnetic layer before an external field is applied, in order to observe domain wall motion through the remaining half of the system.

#### 3.2.1 Ferrimagnetic layer

**Fig. 6:**
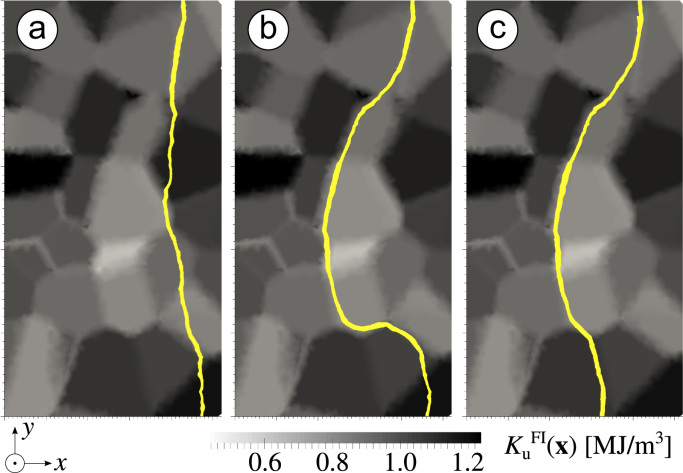
Lateral domain wall movement in a ferrimagnetic film governed by pinning processes with an applied increasing out-of-plane field. The pinning is determined by the distribution of $K_{u,i}^{\mathrm{FI}}$ across the patches. Equilibrium (a), pinning at patches with increased $K_{u,i}^{\mathrm{FI}}$ (b) and depinning in the lower area of (c) are shown.

An investigation of a ferrimagnetic layer reveals that the domain wall is moving laterally and is governed by pinning processes at the patch boundaries. Pinning is caused by the variation of anisotropic properties across the patches as described in Section 2.2. The spatial variation of the magnetic anisotropy constant $K_{u}^{\mathrm{FI}}(x)$ gives rise to energy barriers against domain wall motion at patch interfaces. In Fig. 6 three snapshots of the domain wall movement are depicted. The domain wall is drawn on a *x*–*y*-slice through the ferrimagnet which is coloured by $K_{u}^{\mathrm{FI}}(x)$. The darker the patch appears the higher is its $K_{u,i}^{\mathrm{FI}}$ value.

After artificially setting the domain wall the system is allowed to relax for 2 ns (Fig. 6a) before an external field is applied in *z*-direction. With increased field in Fig. 6b the domain wall gets pushed through patches with weaker anisotropy (light grey) in the centre and stops at the repulsive barrier of patches with increased $K_{u,i}^{\mathrm{FI}}$ (dark grey). The domain wall stays pinned at patch boundaries in the upper and lower regions of the slice. This situation is similar to domain wall pinning in a two-face system being composed of a hard- and a soft-magnetic phase, when the pinning field is proportional to the difference of the magnetocrystalline anisotropy of both phases [27,28]. The repulsive patch can be seen clearly at the bottom region of Fig. 6b, whereas the top part of the domain wall is pinned at a patch outside the presented *x*–*y* plane and therefore cannot be seen. By further increasing the external field, the domain wall is pushed against the repulsive patch at the bottom. When the pinning field $H_{p}=\Delta K_{u}^{\mathrm{FI}}/(2\mu_{0}M_{s})$ [27] is reached, the domain wall spontaneously moves through this patch and pins at the next repulsive patch boundary (Fig. 6c). Each pinning process can be observed as a sudden drop in the magnetization reversal curve.

#### 3.2.2 Ferri-/ferromagnetic bilayer

**Fig. 7:**
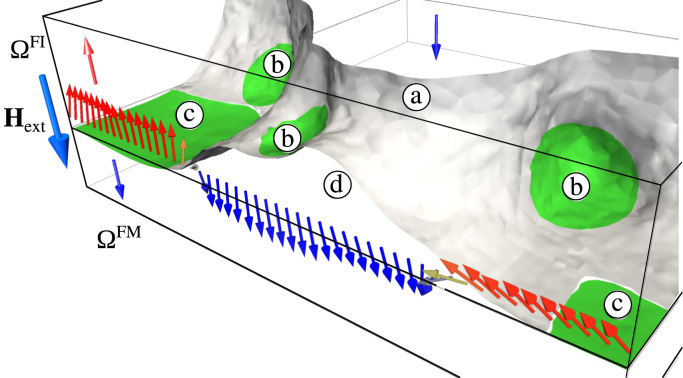
Snapshot of the reversal process with artificially skipped nucleation of $\Omega^{\mathrm{FI}}$. The regions with arrows pointing downwards are already reversed whereas the regions with arrows pointing upwards are still not aligned with the external field $H_{\mathrm{ext}}$. The configuration of the domain wall in the ferrimagnetic phase is determined by pinning at patch boundaries (b) and pinning at the interface of the bilayer system (c). Depinning from patch boundaries (a) and depinning from the interface (d) are both shown in the centre of the $\Omega^{\mathrm{FI}}$ phase.

By adding the ferromagnetic layer the reversal process gets more complex, hence the domain wall position is visualized in three dimensions throughout the reversal process to investigate the partaking mechanisms. A snapshot of this bilayer system is shown in Fig. 7. The domain wall is depicted as a grey surface in the $\Omega^{\mathrm{FI}}$ phase and splits the bilayer system in an already reversed region and a region which is still not reversed. The reversed regions are denoted by the arrows aligned with the external field $H_{\mathrm{ext}}$. The anterior part of the $\Omega^{\mathrm{FI}}$ is still not reversed and is marked by arrows pointing upwards, antiparallel to $H_{\mathrm{ext}}$.

An artificial domain wall is set-up by reversing half of the bilayer, allowing the system to relax to its remanent state and applying an external field. Pinning occurs at two locations: at the interface between the ferrimagnet and the ferromagnetic layer (c) and at patch boundaries (b). The external field exerts a force on the domain wall. In regions without a pinning site the domain wall is pushed further, which leads to bowing of the wall. This is clearly seen at (a) inside the ferrimagnet and at (d) where the wall breaks away from the interface between the ferrimagnet and the ferromagnet. In the bilayer system the exchange coupled ferromagnetic layer helps the reversal by pushing the interface domain wall upwards into the ferrimagnetic phase (d).

## 4 Summary and conclusions

A micromagnetic model for ferrimagnetic thin films according to Mansuripur [11] was adapted and implemented in the 3D finite element micromagnetic package *FEMME*. This model was extended by considering an additional exchange coupled ferromagnetic film. In order to incorporate the microstructural features of the amorphous ferrimagnet, the two layers had to be meshed separately. To restore the continuity at the interface between the two layers, a surface integral technique, as suggested by Dean et al. [24], was used and the interface exchange energy was computed.

The magnetization reversal curve of the ferri-/ferromagnetic bilayer was computed by averaging over many curves using a different exchange coupling energy. In this way the measured curve of Schubert et al. [17] was reproduced. The result indicates that large regions are weakly coupled and only a minor portion exhibits stronger coupling.

The domain wall motion is investigated in a ferrimagnetic film and a ferri-/ferromagnetic bilayer. Both models show a lateral movement of the domain wall, governed by repulsive pinning at the patch boundaries of the ferrimagnetic layer, due to its spatial distribution of anisotropic properties. In the bilayer system pinning occurs also at the interface, but the coupled ferromagnetic layer helps the reversal of the bilayer by pushing the domain wall upwards into the ferrimagnetic layer. These results support the findings of Schubert et al. [17].

## Figures and Tables

**Table 1:** Intrinsic properties of the ferri-/ferromagnetic heterostructure at 70 K.

Phase		$A_{x}\;(\mathrm{pJ}/m)$	$K_{u}\;(\mathrm{kJ}/m^{3})$	$J_{s}\;(\mathrm{mT})$
$\Omega^{\mathrm{FI}}$	Fe_81_Tb_19_	1	889	135
$\Omega^{\mathrm{FM}}$	[Co/Pt]	2	147	628

## Appendix A Supplementary material

Video 1 This video is an extension to Fig. 6 and shows the same data but on three slices: in the centre, at the bottom and at the top of the film. An increasing external out-of-plane field (in the *z*-direction) is applied. The patches are coloured by their uniaxial anisotropy constant. The darker the patch appears, the higher is its anisotropy constant. The lateral domain wall movement in a ferrimagnetic film is governed by pinning processes which are determined by the distribution of the anisotropy constants across the patches.
